# Impacts of particulate matter (PM2.5) on the behavior of freshwater snail *Parafossarulus striatulus*

**DOI:** 10.1038/s41598-017-00449-5

**Published:** 2017-04-05

**Authors:** Danny Hartono, Billion Lioe, Yixin Zhang, Bailiang Li, Jianzhen Yu

**Affiliations:** 1 Department of Environmental Science, Xi’an Jiaotong Liverpool University, 111 Ren’ai Road, Dushu Lake Science and Education Innovation District, Suzhou, 215123 Jiangsu Province P. R. China; 2 XJTLU Huaian Research Institute of New-type Urbanization, 19 Meigao Road, Wisdom Valley, Huaian, 223005 Jiangsu Province P. R. China; 3 Department of Chemistry, Hong Kong University of Science and Technology, Hong Kong, P. R. China

## Abstract

Fine particulate (PM2.5) is a severe problem of air pollution in the world. Although many studies were performed on examining effects of PM2.5 on human health, the understanding of PM2.5 influence on aquatic organisms is limited. Due to wet deposition, the pollutants ﻿in PM2.5 can enter aquatic ecosystems and affect aquatic organisms. This study tested the hypothesis that PM2.5 will negatively affect the behavior of freshwater snail *Parafossarulus striatulus* (Benson, 1842). Along with PM2.5, a number of components (Al, Pb, and Zn) that are commonly present in PM2.5 were also tested for their effects on the snail's behavior. The snail behavior was scored using the Behavioral State Score (BSS), ranging from 0 (no movement) to 5 (active locomotion and fully extended body). The result shows that high PM2.5 concentration dose (7.75 mg/L) induced a significant decrease in snails’ movement behavior, and such reduced movement. The ﻿﻿same﻿ behavior was also observed for treatments with chemical components related to PM2.5, including aluminum and acidity (pH 5.0). In contrast, a low concentration of PM2.5 (3.88 mg/L), lead, and zinc did not significantly affect snails’ behavior. The results suggest that high PM2.5 deposition in water bodies, associated with acidification and some metals, can have an adverse effect on aquatic organisms.

## Introduction

Particulate matters (PM) are defined as a suspension of fine particles with variable composition that are less than 100 micrometers in diameter^1–3^. Elevated level of PM is caused by large-scale human activities, including extensive fossil-fuel and coal combustion, which is often related to acid rain. PM2.5 refers to particulate matters (PMs) having an aerodynamic diameter of 2.5 micrometers or less^2, 4^. A notably large number of studies have been conducted to determine the effects of PM2.5 exposure on human health, such as significant hazards to the respiratory system^5–10^. However, there are not many papers discussing the toxicology of PM2.5 on animal ecology^4^. Considering huge potential health risks which are associated with PM2.5, it is not implausible to assume that PM2.5 can exert negative effects on other organisms within affected regions^4, 11^. Verma *et al.* ^11^ and Zhao *et al.* ^4^ state that aquatic ecosystem can be very vulnerable to PM2.5 toxicity as pollutants tend to accumulate in water bodies through wet and dry deposition, magnifying its concentration and effects. One study, conducted in 2013 summer rainy period in downtown Beijing, found that the concentration of PM2.5 was negatively correlated to the quantity of accumulated rainfall, showing that PM2.5 reduction was from over 400 μg/m^3^ to 10–30 μg/m^3^ in a period with 1.6 mm precipitation^12^. Its ecological effect stems from the acidification of water bodies through acid rains with low pH value (often <4.5)^13–15^. Some studies have correlated sulfate and nitrate aerosols, which are common anthropological pollutants in PM2.5^16–18^ to a pH decrease in affected water bodies^15^. Acidification of aquatic environment has been proven to negatively impact aquatic ecosystem function and species diversity^19, 20^. Therefore, research on investigating the effects of PM2.5 on aquatic ecosystems and organisms is an important topic of aquatic ecology^21^.

PM2.5 sources in urban areas and their surrounding are primarily anthropogenic; up to 71% originates from emission sources such as biomass burning, vehicular exhaust, as well as secondary sulphate/nitrate formation processes, with sizable geographical and temporal variations, while the remaining portions originate from natural sources such as sea sprays and dust storms^21, 22^. PM2.5 is considerably more toxic than larger PM particles; as they contain acidic chemicals like sulphates as well as metals, such as lead, aluminum, and zinc in an oxidized state^21, 23^. In addition, several studies indicate that due to their small sizes, they are able to penetrate deeper into the respiratory system, potentially leading to a number of adverse physiological damage such as alveolar damage and DNA oxidation^2, 7, 9, 24, 25^. Due to its anthropogenic sources, the composition of any given sample of PM2.5 from one area is likely to be distinct from samples obtained from another area^26^. It leads to different levels of toxicity depending on the nature of its sources. Despite the variability, many samples feature acidic ions like sulphates and nitrates in higher than average concentrations^16^. It has been theorized that acidic precipitation associated with sulphates and nitrates would have negative effects on organisms^15^. In addition, for a number of metallic ions in various PM2.5 samples, metal species of Al, Pb, and Zn are among the most prevalent ones^27^, which have potential deteriorating effects on aquatic ecosystem quality through rainfall and surface runoff^28, 29^. These three metal ions were present in high concentrations in PM2.5 samples sourced from various regions, along with other metals such as Fe, Ca, Na and K^23, 30^.

The standard approach to toxicity assessment has been to employ lethal dose (LD 50) experiments, where the value of the parameter is obtained as the minimum concentration at which half of the test organism is killed after a specific amount of time (usually 48–96 hours)^31, 32^. However, this study used a different approach - behavioral examination, because the results of behavioral observations can be used to evaluate sub-lethal effects caused by environmental toxicity, which cannot be studied using LD 50. Sub-lethal analysis can be more informative than lethal dose experiments, because such approach allows a way to view the effects of the test chemicals on the behavior of the organism, which can have far-reaching ecological consequence^32–36^. To that end, small freshwater snails, *Parafossarulus striatulus*, were used as subjects for the experiment, partially because of their small and practical size, as well as their sensitivity to changes in their environment^37^. Since we expected to see a decline in their activity as a result of PM2.5 exposure, ﻿w﻿e hypothesized that PM2.5 exposure will produce a signif﻿icant impact on the snails’ movement behavior. We also conducted an acid treatment, consisting of sulphuric acid, for simulating the acidic nature of PM2.5 and the presence of acidic ions such as sulphates. Similarly a few metallic ions were also selected, because PM2.5 samples﻿ often contain aluminum, lead and zinc^38^, which have potential negative impact on aquatic ecosystems and biota^2, 28, 29, 39^.We hypothesized that aluminum, zinc, and low pH condition will reduce the activity of the snails, in terms of movement behavior, while lead will induce hyperactivity on the snails^31, 37, 39–45^.

## Results

**Figure 1:**
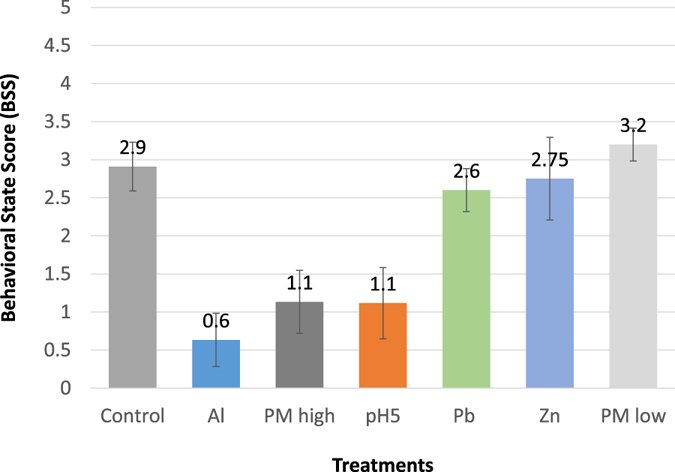
Average BSS score for each treatment. The average values of BSS scores (0–5) at y-axis represent mean ± SE for each treatment (x-axis). High PM2.5, pH 5, and aluminum treatments had significant BSS decrease in snail behaviors. In contrast, lead, zinc, and low PM2.5 did not show significant BSS changes.

In the experiment with 6 different treatments (Table 1) plus control, snails’ behavioral data set consists of six levels of Behavioral State Scores (BSS) (Table 2) for each snail, which translates to 504 individual data entries for 126 individuals (15 for each treatment and 36 for control). All BSS scores from each experimental treatment were averaged and compared with the average of the control group as shown in Fig. 1.

**Table 1:** Summary of the chemicals used in each treatment. There were 6 treatments in this experiment plus a control; two levels of PM2.5 (low and high), three different metals (Al, Pb, Zn), and one level of acidity (pH = 5).

Chemicals	Concentration	Purpose
High PM2.5	2 filter pieces (7.75 mg/L)	Simulate High PM exposure
Low PM2.5	1 filter piece (3.88 mg/L)	Simulate low PM exposure
Aluminum Nitrate	100 μg/L	Test the effect of aluminum
Lead(II) Acetate	200 μg/L	Test the effect of lead
Sulphuric Acid	pH 5	Test pH changes
Zinc Sulphate	100 μg/L	Test the effect of zinc

**Table 2:** Summary of the Behavioral State Score (BSS) criteria. The score ranges from zero to five with zero that is the least active/no movement, while five indicates the highest movement with active locomotion.

Behavioral State Score	Activity correlated
0	Body is retracted and operculum is closed, snail is completely still and hidden.
1	Operculum is ajar but the snail is withdrawn while demonstrating no locomotion.
2	Operculum is ajar but the snail is still withdrawn while allowing their antennae to exit the operculum and moving them about.
3	Snail is more active, the operculum is open and the snail has exposed its head.
4	Snail exposes both head and foot while it conducts movements to re-orient itself.
5	Snail is full extended with its head and foot out while demonstrating active locomotion.

**Figure 2:**
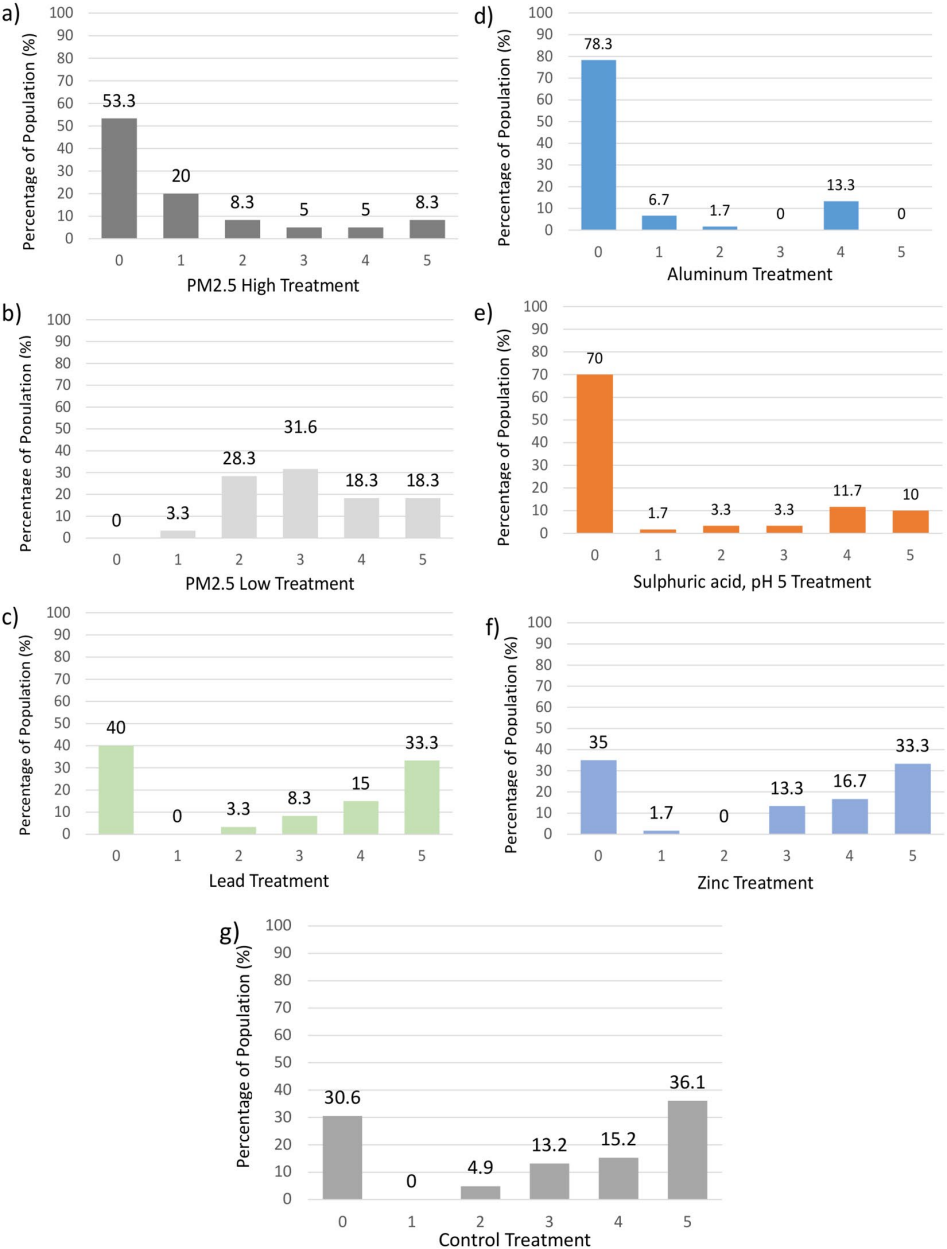
The occurrence distribution (%) of each Behavioral State Score (BSS) from 0 to 5 (x-axis) on each treatment. The y-axis represents the percentage of the sample size that scored a specific category (0–5). For every treatment the total number of snails (n) is 60. The graphs present the results for: (**a**) PM2.5 High Concentration, (**b**) PM2.5 Low Concentration, (**c**) Lead, (**d**) Aluminum, (**e**) Acidity (pH = 5), (**f**) Zinc, (**g**) Control. All setups were performed identically, including the control (a blank consisting of filtered stream water). Treatments of aluminum, acidity (pH = 5), and high PM2.5 concentration had low % values of BSS scores at 1–5.

Overall, the control has an average of BSS 2.91 ± 0.32, pH 5 1.12 ± 0.47, aluminum 0.63 ± 0.35, lead 2.6 ± 0.28, Zinc 2.75 ± 0.54, High PM2.5 1.13 ± 0.41 and low PM2.5 3.2 ± 0.22. Interestingly, the low PM2.5 snail group appeared more active than other treatments (Fig. 1). Figure 2 shows the variation of occurrence distribution (in percentages) for each behavioral score in different treatments. Overall, score zero appears the most often on the high PM2.5, aluminum, and acidity at pH 5 groups. Whereas, score five appears most commonly on the lead, zinc, and control groups. The high percentages of zero score in the high PM2.5, aluminum and acidity at pH 5 treatments indicate their potential effect of toxicity on the snails. Lead and zinc both have high numbers of zero and five scores and averages close to the control average. As such, lead does not seem to have much effect on *P. striatulus*. The low PM2.5 treatment also averages close to the control and even slightly higher, contradicting the high PM2.5 treatment (Fig. 2).

High PM2.5 treatment causes a statistically significant reduction in the snails’ behavior with *p*-value smaller than 0.01 (the Kruskal-Wallis analysis). This is also true for the groups subjected to aluminum and acidity pH 5 solution (*p* values <0.01 and <0.05 respectively). However, this is not the case with low PM2.5 treatment (theoretically about half the amount dissolved in high PM2.5 treatment), which did not induce any significant behavioral change of the snails (*p* > 0.05, *p* = 0.73). Lead and Zinc exposure also failed to show any statistically significant correlation with the snail’s behavior (*p* = 0.71 and 0.86, respectively). The results of the experiments, including p-values obtained in comparison with the controls are displayed in Table 3.

**Table 3:** Result of the non-parametric Kruskal-Wallis statistical analysis. H value shows the discrepancies between the rank sums of BSS from one treatment with the control; higher means more significant. High PM2.5, pH 5, and aluminum shows significant change in the snails’ behavior (*p* < 0.05).

Test Substances	H	*p*-value
PM2.5 High	6.859	<0.01
PM2.5 Low	0.118	0.73
Aluminum Nitrate	14.911	<0.01
Lead(II) Acetate	0.134	0.71
Sulphuric acid (pH 5)	6.463	<0.05
Zinc Sulphate	0.032	0.86

The elemental/ organic carbon (EC/OC) and ionic compound (IC) analysis result is presented in Table 3, in which the highest ion was suphate.

## Discussion

To the extent of the authors’ knowledge, this is the first paper to assess the toxicological effect of PM2.5 on an aquatic organism. The results show that there was a significant decrease of snails’ behavioral state score (BSS) under high concentration of PM2.5 treatment, as well as increased acidity (pH value = 5), the latter of which is the characteristic of PM2.5.

High PM2.5 concentration (approximately 7.75 mg/L) caused significant difference in the behavior of tested snails compared to the control group without PM2.5 treatment. Low PM2.5 exposure results, interestingly, are not statistically different from the control results, but instead seem to exhibit higher scores on average. This could mean that low PM concentrations may cause some hyperactivity. A possible explanation for this phenomenon is that the components PM2.5 exist in different forms (species) which result in different bioavailability and solubility^46^. The toxicity of the components in low PM treatment may still be below the toleration limit of the snails. On the other hand, when the concentration of the PM was doubled in the high PM treatment, the toxicity level exceeded the limit.

From the BSS spread graphs, we can see that the groups with statistically significant results have more zero scores than the other treatments. The aluminum treatment result correspond to White *et al.* ^47^ who found that aluminum toxicity merely affected snail as sub-lethal behavioral depression. It is unknown if the effect would cease after a longer time since our experiment focused on short term effects. Interestingly, the shapes of the lead and zinc graphs are similar to that of the control group, which imply that, despite their proven toxicity on humans, they seem to be less dangerous for *Parafossarulus striatulus.* In an experiment by Truscott *et al.* ^23^, lead was proven to induce hyperactivity on the snail *Lymnaea stagnalis* in short-term testing (up to 19 hours). Compared to his research, our experiment did not show any hyperactivity behavior (the average score is almost the same compared with the control group). However, this might be caused by the higher limit of the Behavioral State Score as theorized earlier. The results of the zinc test are supported by prior research on zinc toxicity to a different freshwater snail species (*Lymnaea* spp., *Physa* spp.). Zinc was found to be the least toxic among some of the more common heavy metal contaminants^31, 37, 48^. It should be noted; however, the types of zinc compounds and parameters differ from ones used on this experiment.

Other studies had found that PM2.5 constituents generally make it acidic, which is the reason using sulphuric acid to increase acidity in one of the treatments^23^. Our results at pH 5 seem to show a strong correlation between low pH and reduction in snail’s activity. Preliminary tests with lower pH value of 4 showed immediate withdrawal, supporting the hypothesis. Further it is interesting to note that while sulphuric acid incited a clear response, zinc sulphate did not have a similar effect. This can potentially means that the snails were responding to the H^+^ cations instead of sulphate anions in the acid experiment^49^.

One point of contention is the method of PM2.5 dispersal. PM2.5 treatment was introduced into the experimental setup by placing 6 mm circular cutouts of the glass fiber filter the PM was collected on into the solution inside the petri dish. This method – although showing some significant effects – is not foolproof; most of the PM2.5 remained embedded in the glass fiber mesh and as a result only some fraction of the PM2.5 was released into the water. In comparison, the concentration presented in this experiment was based on the assumption that the PM2.5 was diluted entirely. Nonetheless the high PM test still resulted in a statistically significant change in behavior relative to controls. However, this still causes some problems for calculation as the exact amount of PM2.5 released into the solution cannot be obtained with high precision. Therefore, the quantity of PM2.5 in this report is likely to be slightly exaggerated compared to the actual amount that the snails are exposed to.

Future studies should consider examining long-term effects of those treatments, and effects of chronic PM2.5 (and its constituents) exposure on aquatic organism behavior and population dynamics. Continuing long term effects, studies on the seasonal composition and concentration of PM2.5 have consistently found that summer time concentrations are low relative to other seasons. As such, similar experiments﻿ can be conducted with PM2.5 samples collected from other seasons to determine differences in effects based on seasonal variation^50, 51^. Lastly, while this experiment showed that PM2.5 can be harmful to aquatic organisms, it did not provide detail information of which particular components of PM2.5 contributes to its toxicity or whether there are antagonistic, additive, synergistic, or potentiative interactions; as such, a full assay of its components will be beneficial for understanding of machnisms.

## Conclusion

PM2.5 is an important pollutant composed of a complex mixture of geographically and temporally variable components. Despite the variability, anthropogenic PM2.5 consistently contains hazardous chemicals such as heavy metals and acidic anions. The results of the study indicated: (i) the snails tested did react adversely to PM2.5 and appeared to have a threshold for how much PM2.5 they can be exposed to, (ii) among the chemicals tested, pH 5 sulphuric acid had the largest impact on the snails, where the Behavioral State Scores were the lowest across all treatments. (iii) the scores under﻿ aluminum treatment were significantly lower compared to the controls, while scores from the other metal ions tested were not statistically significant. In conclusion, PM2.5 exposure were found to have a negative impact on freshwater snails. Furthermore, of the chemicals hypothesized to exist within PM2.5, sulphuric acid aerosols and aluminum had the most negative effects on snail behavior.

## Methods

### Snail Maintenance

The freshwater snails *Parafossarulus striatulus* (Benson, 1842) were collected from a stream channel close to Xi’an Jiaotong-Liverpool University (31°16′18.025″N, 120°44′10.687″E). *Parafossarulus striatulus* was selected as the subject of the experiment due to its abundance in the natural streams and lakes. Its size is also practical. The average shell length was 6 ± 1 mm and therefore, was advantageous considering the limited amount of PM2.5 which is relatively difficult to obtain in large quantities. In addition, freshwater snails tend to be sensitive to environmental changes which make them suitable for this experiment^37^. Following collection, the snails were then randomly selected based on size, specifically, specimens of about 6 ± 1 mm.

The stream water was also fetched and then filtered using Whatman® double-ring qualitative filter paper with 11-micron pore size and 9 cm diameter in order to remove suspended food particles. It was later used for the starving period and the experiment sessions.

### PM2.5 Collection and Experimental Treatments

Prior to the experiment, PM2.5 particles were collected using a DUSTTRAK™ II unit manufactured by TSI (3.0 L/min flow rate) in conjunction with glass fiber filters (ø 37 mm, 0.3 μm pore size, 99.995% efficiency). The collection site was at an apartment balcony (31°16′43.4″N, 120°44′44.7″E). The device was run for several weeks with filters being replaced about once a week on average. The exposed filters were then stored in a freezer with a temperature of −20 °C to preserve the chemical composition. Immediately afterwards, the filters were measured gravimetrically using a 10^−4^ gram accuracy balance. Since extracting the PM directly by rinsing and stirring the filter paper in the water destroyed the glass fiber itself, we decided to just place a small part of the filter in the experiment petri dishes. The PM2.5 on the filter paper weighed about 0.0047 g on average. A 6 mm-diameter-hole puncher was used to cut the glass fiber into smaller circles. The center point (about 2 mm diameter) was ignored because it was visibly darker than the surrounding area, indicating higher concentration. This resulted in each circle containing approximately 155 μg PM2.5 under the assumption that all the PM2.5 were diluted into the water. The high PM treatments were simulated with two circles, while low PM2.5 treatments only used one. Consequently, the PM2.5 concentrations in these two treatments were approximately 7.8 mg/L and 3.9 mg/L, respectively.

Four additional treatments that are associated to PM2.5 were conducted to test their effects on behavior of freshwater snails. The first one was aluminum in form of aluminum nitrate with the concentration of 100 μg/L. Secondly, we experimented using zinc in form of zinc sulphate with the same concentration as aluminum. Next, the effect of lead was also examined in form of lead (II) acetate with higher concentration of 200 μg/L. All the chemicals used were analytical grade. The last treatment was acidity (pH value at 5)^14^. The pH 5 solution was obtained by diluting high concentration sulphuric acid, our choice of sulphuric acid is representative of sulphate pollution that causes mildly acidic rains^23^. Each treatment was replicated five times.

A separate filter, which was different one from the one used for snail behavior tests, was analyzed for elemental/organic carbon (EC/OC) analysis followed by ionic compound (IC) analysis, to examine the components present in PM2.5 collected at this location. For carbon analysis, a 1 cm^2^ punch of the filter was removed and analyzed with thermal/optical transmittance method using Aerosol OCEC analyzer. For IC analysis, half of the filter was extracted by 5 ml of distilled de-ionized water, followed by the utilization of an Ion chromatograph (Table 4).

**Table 4:** Concentration of ions, organic carbon (OC), and elemental carbon (EC) in a PM2.5 sample. The concentrations are presented in micrograms per area of the filter (cm^2^). The ions with the highest concentration were sulphate, nitrate, and sodium ions.

Species	Concentration (μg/cm^2^)	Species	Concentration (μg/cm^2^)
Na^+^	37.54	NO_3_^−^	16.92
NH_4_^+^	1.01	SO_4_^−^	71.54
K^+^	1.80	C_2_O_4_^2−^	2.35
Mg^2+^	0.57	OC	12.5
Ca^2+^	4.57	EC	9.37
Cl^−^	4.91		

### Experimental Procedure

**Figure 3:**
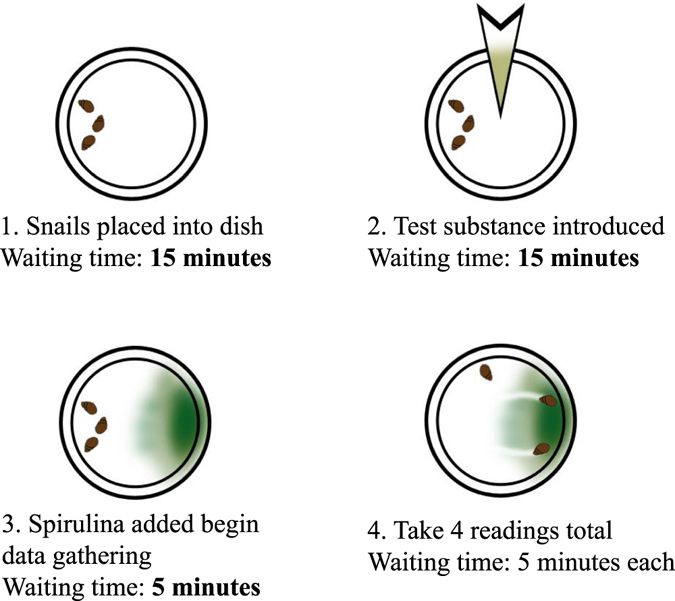
Illustration of the methodology used to test snails’ movement and behaviors. Step 1: three snails were put on every dish and provided 15 minutes acclimation time. Step 2: the test substances were introduced and another 15 minutes of time was given as an acclimation period. Step 3: spirulina was added. Step 4: after 5 minutes, 4 readings were taken every 5 minutes.

Fresh snails were collected one day prior to each experiment session. They were isolated and starved for approximately 24 hours in a container filled with filtered water from the channel. After that they were randomly selected, cleaned a little bit, and measured. When the length was outside the range (6 ± 1 mm), it was discarded and following that, another one was selected. The experimental setup consisted of 7 petri-dishes (diameter = 11 cm); 5 of which were treatment replicates along with 2 controls. Each petri dish was filled with 40 ml of filtered pond water and three snails were placed into each petri dish giving a total of 21 in each session. Every session took approximately 50 minutes to complete; the first 15 minutes was designated as acclimation time to allow the snails to adapt to their new surroundings. Once the acclimation time ended, the tested substances were introduced into the 5 dishes of the experimental group, another 15 minutes were then given before we pipetted in Spirulina algae that can attract snails as their food. After the spirulina algae had been added, a survey of the snails’ activities was performed every 5 minutes for 20 minutes; taking readings of four data points for every snail (Fig. 3), which used scores (0–5) that are associated with different behaviors (the Behavioral State Score, Table 2).

The behavioral parameter was measured with the Behavioral State Score (BSS)^52^. This scoring system is similar to an experiment performed by other researchers^43, 52, 53^. The system works by scoring the snails’ level of activity from 0 to 5, which are based on their observed behavior: (i) 0 points for full retraction into its shell, (ii) 1 point for being withdrawn, (iii) 2 points for antennae movement only, (iv) 3 points for a protruding head without movement, (v) 4 points for an extended foot and head with oriented movement, and (vi) 5 points were given for active locomotion, and ref. 52. Table 2 shows the different scores and the behaviors they are associated with.

### Statistical Analysis

Statistical analysis was performed through the use of a non-parametric Kruskal-Wallis test with the level of significance set at *p* = 0.05 and a degree of freedom of 1^43^. Non-parametric statistical analysis method was used due to the categorical nature of BSS, where each number represents a distinct state of being for each individual instead of a gradual scale. All the data were represented in means ± standard error.
